# Immersion Ultrasound Therapy in Combination with Manual Therapy in the Treatment of Ischemic Digital Ulcers in Systemic Sclerosis

**DOI:** 10.3390/medicina59071335

**Published:** 2023-07-20

**Authors:** Dalila Scaturro, Antimo Moretti, Fabio Vitagliani, Giuliana Guggino, Sofia Tomasello, Davide Lo Nardo, Lorenza Lauricella, Giovanni Iolascon, Giulia Letizia Mauro

**Affiliations:** 1 Department of Surgical, Oncological and Stomatological Disciplines, University of Palermo, 90127 Palermo, Italy; giulia.letiziamauro@unipa.it; 2 Department of Medical and Surgical Specialties and Dentistry, University of Campania “Luigi Vanvitelli”, 80138 Naples, Italy; antimo.moretti@unicampania.it (A.M.); giovanni.iolascon@unicampania.it (G.I.); 3 Faculty of Medicine and Surgery, University of Catania, 90121 Catania, Italy; fabiovitagliani93@libero.it (F.V.); davidelon.991@gmail.com (D.L.N.); 4 Rheumatology Section, Biomedical Department of Internal Medicine, University Hospital “P. Giaccone”, 90127 Palermo, Italy; giuliana.guggino@unipa.it; 5 Faculty of Medicine and Surgery, University of Palermo, 90100 Palermo, Italy; sofia.tomasello@hotmail.com; 6 Paolo Giaccone University Hospital, 90127 Palermo, Italy; lorenzalauricella@libero.it

**Keywords:** systemic sclerosis, rehabilitation, skin ulcer, ultrasound therapy, disability

## Abstract

*Background and Objectives*: Digital ulcers (DUs) are the most common complication in patients with Systemic Sclerosis (SSc). They cause pain with hand dysfunction and negatively impact activities of daily and working life. Our study aims to evaluate the efficacy of a combined treatment of manual therapy and ultrasound therapy in SSc patients with ischemic DU (IDU) compared to manual therapy alone. *Materials and Methods*: We conducted a before-and-after study (non-randomized study). We enrolled a consecutive series of IDU patients undergoing rehabilitation treatment and divided them into two groups: a treatment group consisting of patients undergoing a combination of manual therapy and US water immersion and a standard care group consisting of patients subjected to manual therapy alone. At the time of the first visit (T0) and at the end of the 4-week rehabilitation period (T1), we evaluated functional capacity, pain intensity, ulcer evolution, and quality of life. *Results*: In the treatment group, we observed a statistically significant improvement in the functional capacity of the hand (DHI: 28.15 ± 11.0 vs. 19.05 ± 8.83; *p* < 0.05), pain (NRS: 5.55 ± 1.2 vs. 2.9 ± 1.09; *p* < 0.05), and PSST score (24.4 ± 4.0 vs. 16.2 ± 2.36; *p* < 0.05). In the standard care group, we observed a statistically significant improvement only for the functional capacity of the hand (DHI: 28.85 ± 9.72 vs. 22.7 ± 7.68; *p* < 0.05). Finally, from the comparison between the treatment group and the standard care group, we observed statistically significant improvements in pain (2.9 ± 1.09 vs. 4.5 ± 1.07; *p* < 0.05) and in the PSST scale (16.2 ± 2.36 vs. 20.4 ± 4.02; *p* < 0.05). Furthermore, at the end of treatment in the treatment group, 15 ulcers (62.5%) were completely healed, while in the standard care group, only 3 ulcers were completely healed (14.3%). *Conclusions*: Combined treatment with manual therapy and ultrasound therapy appears to be useful in the management of IDU in patients with scleroderma.

## 1. Introduction

Systemic sclerosis (SSc) is a chronic autoimmune disease characterized by vascular disease, inflammation, immune response, and accumulation of collagen in the skin and other organs resulting in fibrosis [1,2]. This condition leads to an alteration of tissue architecture with consequent loss of organ function in the terminal stages of the disease [3].

The etiology is still uncertain, but an important role in its development seems to be played by genetic and environmental factors [2]. It has an incidence of between 4 and 43 million people per year and mostly affects women [4,5,6,7].

According to the new 2017 criteria of the SSc classification of the American College of Rheumatology (ACR) and the European League Against Rheumatism (EULAR) [8], skin thickening of the fingers is sufficient to make a diagnosis. In addition, there are seven other typical pathological conditions, including fingertip ulcers or pitting scars, localized finger telangiectasias, capilloscopic changes, pulmonary hypertension associated with digital ulcers (DUs), Raynaud’s phenomenon, and the presence of autoantibodies [8,9].

Thickening and fibrosis of the skin and internal organs, caused by progressive vascular disease, are the main clinical manifestations [2,3]. Involvement of the hands is responsible for a marked disability in these patients due to the presence of swollen fingers, DUs, cutaneous sclerosis, calcinosis of the skin, and pruritus [10,11].

DUs are the most common complication, affecting over 50–70% of patients with SSc. They are recurring lesions affecting hands and feet, characterized by loss of continuity and depth of the skin. They are caused by ischemic vessel damage and are indicators of overall disease severity and organ involvement [10,12]. DUs often lead to pain with hand dysfunction and have a negative impact on activities of daily living and work, resulting in disability [10]. Furthermore, the presence of DUs requires continuous follow-up to prevent possible complications, such as infections that can involve both the skin and underlying tissues, such as bone, resulting in osteomyelitis [12,13].

Depending on the extent of skin involvement, systemic sclerosis can be divided into a limited cutaneous form (lcSSc), with involvement limited to the skin of the hands, forearms, feet, and face, and a diffuse cutaneous form (dcSSc), involving skin proximal to the elbows and trunk [1].

Currently, the treatment of SSc focuses on various pharmacological and non-pharmacological interventions based on recommendations and evidence published by the EULAR and EULAR Scleroderma Trials and Research (EUSTAR) groups [8]. To date, there are no specific treatments for the treatment of DUs. Several systemic treatments are available [14,15,16,17,18,19], such as immunomodulators [8], UVA phototherapy [16], topical calcitriol [17], and retinoids [18]. In addition, local therapies with good aesthetic and functional results can also be used, such as the injection of autologous fat and platelet-rich plasma (PRP) [19,20] or adipose-derived stromal cells (ADSC) combined with hyaluronic acid (HA) [21] or HA and PRP [21,22]. Finally, physical modalities for DUs have also been proposed, including ultrasound therapy [23,24,25] and connective tissue massage, mainly using the McMennel technique [26].

Our study aims to evaluate the efficacy of ultrasound therapy in combination with manual therapy in the management of systemic sclerosis patients with IDU in terms of hand functional capacity, pain, wound healing, and quality of life.

## 2. Materials and Methods

This is a before-and-after study (non-randomized trial) conducted at the Functional Recovery and Rehabilitation Unit of the A.O.U.P. Paolo Giaccone in Palermo, in collaboration with the Rheumatology Unit of the same hospital. Patients with scleroderma presenting IDU were consecutively enrolled (from April 2022 to November 2022).

All aspects of the study were reviewed and approved by the local “Palermo 1” ethics committee, with reference number 6/2020. The ethical guidelines of the Declaration of Helsinki were followed for the study, and the information was handled following the guidelines of the Good Clinical Practice (GCP). The study was registered with ClinicalTrials.gov (NCT05907200).

### 2.1. Patients

Inclusion criteria were a diagnosis of SSc according to the ACR and EULAR criteria [8], the presence of IDU in the active phase, naïve to rehabilitation treatment for their hands and upper limbs, and capacity to provide informed consent. In both groups, all patients continued their usual pharmacological treatments (alprostadil-α-cyclodextran, calcium channel blockers, topical glyceryl trinitrate, proton pump inhibitors, clebopride, steroids, cyclophosphamide, azathioprine, D-penicillamine, and methotrexate).

Exclusion criteria were the presence of skin lesions due to other conditions (e.g., trauma), pregnancy, infectious diseases (e.g., HIV, HBV, HCV), myositis, arthritis, other rheumatological diseases, and immunodepression.

Using our hospital’s database, we enrolled a consecutive series of patients with IDU who had undergone rehabilitation treatment and met our inclusion criteria.

### 2.2. Intervention and Control

Based on the type of rehabilitation treatment received, the patients were divided into two groups: a treatment group consisting of patients who underwent rehabilitation treatment consisting of a combination of manual therapy and water immersion US and a standard care group consisting of patients who underwent rehabilitation treatment consisting of manual therapy alone [26]. The rehabilitation treatment was performed by all patients at the same time of year (from April to June) and was carried out for 20 sessions daily, 5 days a week, for 4 consecutive weeks. Some clinical information and educational recommendations on skin nutrition and protection were provided to all patients.

### 2.3. Description of Manual Therapy Techniques

The proposed manual therapy lasted 90 min and involved a combination of three different techniques: McMennel manipulation, connective tissue massage, and the Pompage mobilization technique [26].

McMennel manipulation is a technique for reducing joint stiffness and reducing pain by stretching the capsuloligo-mental complex. It was used for 40 min. It prevents the development of claw deformity and increases the trophism of the cartilage, resulting in an improvement in hand mobility and the extrinsic strength of the hand muscles, decreasing joint pain and stiffness [27].

Connective tissue massage (lasting 30 min) improves the blood circulation of the skin and plays a muscle-relaxing role, keeping the skin soft [28].

The Pompage mobilization technique lasts 20 min and consists of slow and progressive mobilizations through a rhythmic and regular movement of traction and release, which allows the recovery of the physiological length of the soft tissues [28].

### 2.4. Description of Therapeutic US Technique

Patients in the treatment group also received treatment with a US (UT2 CE0476 certified I-Tech medical device). The USs were used with a frequency of 1 MHz, an intensity of 1 W/cm^2^, a duty cycle of 60%, and a duration of 15 min per session. The patient’s hands were immersed inside a metal container with a diameter of 90 cm, containing 4 L of water at a temperature of 37–37.5 °C. An emitter handpiece with radiant air of 5 cm^2^ was then inserted inside the container, about 2 cm from the body surface. The action of therapeutic ultrasound reduces inflammation and bacterial counts on wounds and improves cell proliferation and neoangiogenesis [23,24].

### 2.5. Outcome Measures

Demographic, anthropometric, and clinical data were collected at baseline. Height and weight were measured in each subject, and BMI was then calculated. At the time of the first visit (T0) and the end of the 4 weeks of rehabilitation (T1), the same expert physiatrist (D.S.) subjected the patients to the following evaluation scales: numerical evaluation scale (NRS) [29] for ache; Duruoz hand index (DHI) [30] for functional ability; Pressure Sore Status Tool (PSST) [31] for ulcer healing; and Short Form Health Survey 36 (SF-36) scales [32] for quality of life (QoL). Our primary outcome measure was functional capacity (DHI), while our secondary outcome measures were pain (NRS), ulcer healing (PSST), and QoL (SF-36).

The DHI is a self-reported questionnaire designed to assess hand activity limits. It includes 18 items that are rated on a 6-point scale where 0 is “without difficulty”, and 5 is “impossible” [30]. The PSST includes 13 items relating to the characteristics of the wound and surrounding tissue, which are assigned a score from 1 to 5. The total sum of all scores provides the PSST score, where the higher the final scores, the more severe the state of the lesion will be [31]. The SF-36 is a questionnaire comprising eight multiple-choice questions that can be divided into two large subgroups: the physical component of the disease and the mental component of the disease. A score is assigned to each scale; the higher the score, the better the state of mental and physical health. The score ranges from 0 (worst state of health) to 100 (best state of health) [32].

### 2.6. Statistical Analysis

All analyzes were performed using R software (R Core Team, 2013). The sample size was calculated to detect a difference in DHI of 5.1 units and a loss of follow-up of 20%. The estimated sample size calculation was 24 for each group. For the comparison of the quantitative variables, we used the t-test, and for the ordinal variables, Mood’s median test. Values < 0.05 were considered statistically significant.

## 3. Results

**Figure 1:**
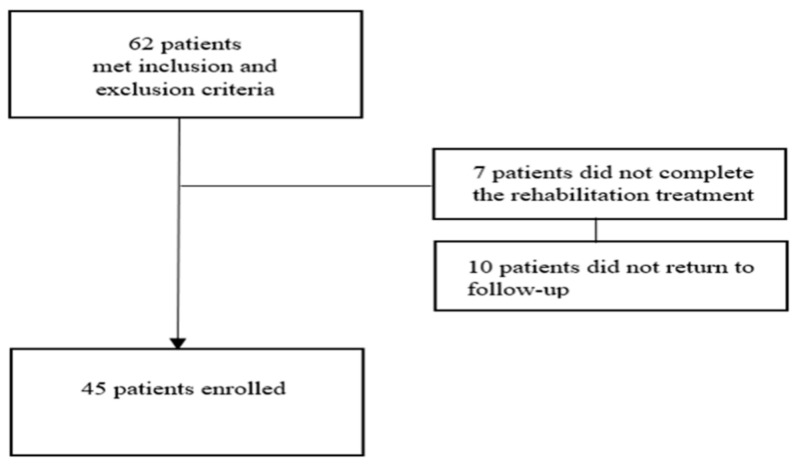
Patient recruitment.

Sixty-two scleroderma patients with IDU were considered in this study. Of these, 7 had not completed the proposed rehabilitation treatment, and 10 did not present themselves for follow-up at the end of the 4-week rehabilitation treatment. A total of 45 patients were included in the study, 24 patients belonging to the treatment group and 21 patients belonging to the standard care group (Figure 1).

The included patients had a mean age of 61.12 ± 8.83 years and included 33 (73.3%) women and 12 (26.7%) men, with a mean BMI of 24.6 ± 2.11 kg/m^2^. Thirteen patients (32.5%) had pulmonary involvement (interstitial lung disease and/or pulmonary hypertension). All patients had ulcers on their fingertips and presented stiffness and loss of joint function due to flexion contractures caused by skin retraction. The included patients had a mean DHI value of 28.5 ± 10.4, with a mean perceived pain of 5.52 ± 1.22 points on the NRS. Finally, the mean value of the PSST scale was 24.32 ± 4.14, and of the SF-36 scale, 57.2 ± 7.98. No statistically significant between-group difference in baseline characteristics was reported (Table 1).

Table 2 shows the effects of the combined rehabilitation treatment of immersion ultrasound therapy and manual therapy in the treatment group at T1. We observed statistically significant improvements in functional capacity (28.15 ± 11.0 vs. 19.05 ± 8.83; *p* < 0.05), pain (5.55 ± 1.2 vs. 2.9 ± 1.09; *p* < 0.05), and PSST score (24.4 ± 4.0 vs. 16.2 ± 2.36; *p* < 0.05). Finally, the QoL did not significantly change at follow-up (SF-36: 57.05 ± 9.1 vs. 52.0 ± 8.75; *p* = 0.08) (Table 2).

Table 3 shows the effects of manual therapy alone in the standard care group at T1. A statistically significant improvement was observed only for the functional capacity of the hands (28.85 ± 9.72 vs. 23.7 ± 7.68; *p* < 0.05). No statistically significant improvement was observed for pain (5.5 ± 1.24 vs. 4.5 ± 1.07; *p* = 0.08), PSST scale (24.25 ± 4.27 vs. 20.4 ± 4.02; *p* = 0.16), and quality of life (57.35 ± 6.66 vs. 54.5 ± 6.54; *p* = 0.18) (Table 3).

Finally, in Table 4, we compare the results obtained in the treatment group and the standard care group at T1. From the comparison, in the treatment group compared to the standard care group, we observed statistically significant improvements in pain (2.9 ± 1.09 vs. 4.5 ± 1.07; *p* < 0.05) and in the PSST scale (16.2 ± 2.36 vs. 20.4 ± 4.02; *p* < 0.05) (Table 4). Furthermore, at the end of the treatment in the treatment group, 15 ulcers (62.5%) were completely healed, while in the standard care group, only 3 ulcers were completely healed (14.3%).

## 4. Discussion

IDUs are common complications in patients with scleroderma [33]. They are very painful lesions with slow healing that lead to a great deal of disability in affected patients. It is estimated that 15–25% of patients with SSc have active IDUs [34]. In this study, we aimed to compare the effectiveness of two different rehabilitation treatments in the management of IDUs of patients with scleroderma in terms of functional capacity. In addition, the effects of these interventions on pain, ulcer healing, and QoL were also compared.

Our results showed a significant improvement in the functional capacity of the hand in both groups. This finding emphasizes the importance of using manual therapy in combination with pharmacological treatments for the management of IDU in patients with scleroderma. SSc patients with IDU have reduced hand mobility and increased global and hand disability compared to those without IDU [35]. Rehabilitation treatment appears useful in reducing the impact of hand impairments in patients with SSc. Boggi et al. [26] demonstrated that the combination of connective tissue massage and McMennell’s joint manipulation proved to be more effective than a program based on daily exercises performed at home in treating the hands of patients with scleroderma. At the end of the treatment period, the authors observed an increase in the HAMIS test and the Cochin Hand Functional Disability Scale, as well as an improvement in mobility, fine movements, and hand function [26]. On the other hand, Mugii et al. [36] studied the effect of self-administered finger lengthening in patients with SSc, showing an improvement in the range of motion in each finger after as early as one month. The authors demonstrated that the combination of self-stretching exercises can be useful for maintaining the efficacy achieved with a rehabilitation program supervised by a physiotherapist. In a quasi-experimental study, the exercise demonstrated positive effects on isometric muscle strength, muscle function, and hand functional capacity in SSc-related IDU. However, it has shown little effect on the healing of IDU or Raynaud’s phenomenon [37].

Another finding highlighted in our study was that significant pain relief was obtained only in the group that received a combination of manual therapy and therapeutic US, compared to patients treated with manual therapy alone. Pain management is of paramount importance in patients with IDUs. Moreover, in patients with systemic sclerosis, non-steroidal anti-inflammatory drugs (NSAIDs), despite their efficacy, should be avoided in favor of paracetamol and opiates due to their known vascular effects. The cause of pain in IDUs is tissue ischemia [35], and the pain relief observed in our study could result from the thermal effect of US. This method, by means of mechanical vibrations, transfers energy to the tissues, inducing the dilation of blood vessels and increasing cell metabolism through the supply of oxygen and nutrients. In addition, the thermal effect of US also has an analgesic action, resulting in changes in pain threshold and tissue viscoelasticity [38,39].

Our study also suggests that ulcer healing seems to be better in the group of patients undergoing the combined treatment of manual therapy and therapeutic US, compared with the standard care group. In addition to the vasodilation induced by US, the mechanical action of this intervention might positively affect the wound tissue by promoting and stimulating cell proliferation [40]. This finding is in line with a previous case report of a patient with a large, painful, and infected DU that was treated with low-intensity US three times a week for 5 min. After 8 weeks from the start of treatment, a decrease in pain score from 10 to 0 was observed, and complete wound closure after 10 weeks [40].

Finally, although a greater improvement in SF-36 score was observed in the treatment group, no significant improvement in quality of life was reported in any group. However, this finding should not be surprising as the main difficulties patients with systemic sclerosis complain about are in activities of daily living (ADLs), including walking, cleaning, and sports training [36]. Hence, although most ADLs involve the use of the hands, the major limitations in these activities are likely related to other disease-related complications [36]. The strength of our study is that it is the first comparison between two different rehabilitation methods in the management of IDU. Our previous study focused on evaluating the effectiveness of the combined rehabilitation treatment of manual therapy and therapeutic US on patients with IDU but lacked a standard care group. However, there are several limitations to this study. First, the study design increases the risk of bias. Furthermore, two further limitations are the short follow-up period which, however, would have been influenced by seasonality, the lack of assessment of the frequency of relapses, and the efficacy of the treatment over time.

Furthermore, we did not make the comparison with a control group because the ethics of our department requires that all subjects with IDU undergo therapy. Finally, a rare disease causes a small sample.

## 5. Conclusions

In patients with systemic sclerosis, the association between ultrasound and manual therapy can be considered effective treatment for IDU, pain, and hand motility. It can be said that ultrasound therapy is safe even if there are ulcers. Manual therapy is essential to improve motility and for carrying out activities.

## Figures and Tables

**Table 1:** General characteristics at baseline.

Characteristics	Total (*n* = 45)	Treatment Group (*n* = 24)	Standard Care Group (*n* = 21)	*p*-Value
Age, mean ± SD	61.12 ± 8.83	61.05 ± 9.3	61.2 ± 8.34	0.96
Sex, n° (%)				
Male	12 (26.7)	7 (29.1)	5 (23.8)	0.88
Female	33 (73.3)	17 (70.9)	16 (76.2)	
BMI (kg/m^2^), mean ± SD	24.6 ± 2.11	24.3 ± 1.88	25.1 ± 1.55	0.56
DHI, mean±SD	28.5 ± 10.4	28.15 ± 11.0	28.85 ± 9.72	0.82
NRS, mean ± SD	5.52 ± 1.22	5.55 ± 1.2	5.5 ± 1.24	0.89
PSST, mean ± SD	24.32 ± 4.14	24.4 ± 4.0	24.25 ± 4.27	0.90
SF-36, mean ± SD	57.2 ± 7.98	57.05 ± 9.1	57.35 ± 6.66	0.65

DHI: Duruoz Hand Index; NRS: Numerical evaluation scale; PSST: Pressure Sore Status Tool; SF-36: Short Form Health Survey 36.

**Table 2:** Effect of combined treatment of immersion ultrasound therapy and manual therapy in IDUs in the treatment group.

Characteristics	T0	T1	*p*-Value
DHI, mean ± SD	28.15 ± 11.0	19.05 ± 8.83	<0.05
NRS, mean ± SD	5.55 ± 1.2	2.9 ± 1.09	<0.05
PSST, mean ± SD	24.4 ± 4.0	16.2 ± 2.36	<0.05
SF-36, mean ± SD	57.05 ± 9.1	52.0 ± 8.75	0.08

DHI: Duruoz Hand Index; NRS: Numerical evaluation scale; PSST: Pressure Sore Status Tool; SF-36: Short Form Health Survey 36.

**Table 3:** Effect of manual therapy in IDUs in the standard care group.

Characteristics	T0	T1	*p*-Value
DHI, mean ± SD	28.85 ± 9.72	23.7 ± 7.68	<0.05
NRS, mean ± SD	5.5 ± 1.24	4.5 ± 1.07	0.08
PSST, mean ± SD	24.25 ± 4.27	20.4 ± 4.02	0.16
SF-36, mean ± SD	57.35 ± 6.66	54.5 ± 6.54	0.18

DHI: Duruoz Hand Index; NRS: Numerical evaluation scale; PSST: Pressure Sore Status Tool; SF-36: Short Form Health Survey 36.

**Table 4:** Comparison between the treatment group and the standard care group at T1.

Characteristics	Treatment Group	Standard Care Group	*p*-Value
DHI, mean ± SD	19.05 ± 8.83	23.7 ± 7.68	0.07
NRS, mean ± SD	2.9 ± 1.09	4.5 ± 1.07	<0.05
PSST, mean ± SD	16.2 ± 2.36	20.4 ± 4.02	<0.05
SF-36, mean ± SD	52.0 ± 8.75	54.5 ± 6.54	0.29

DHI: Duruoz Hand Index; NRS: Numerical evaluation scale; PSST: Pressure Sore Status Tool; SF-36: Short Form Health Survey 36.

## Data Availability

Data used to support the findings of this study are available from the corresponding author upon request.
